# Management of early-stage breast cancer patients during the coronavirus disease 2019 (COVID-19) pandemic: The experience in China from a surgical standpoint

**DOI:** 10.7150/jca.50501

**Published:** 2021-02-22

**Authors:** Wan Wang, Baoliang Guo, Chunguo Cui, Tong Sun, Shengnan Liu

**Affiliations:** 1 Department of Breast Surgery, China-Japan Union Hospital of Jilin University, 126 Xiantai Blvd, Changchun 130033, China.; 2 Department of General Surgery, The Second Affiliated Hospital of Harbin Medical University, 246 Xuefu Street, Harbin 150001, China.

**Keywords:** Early-stage breast cancer, COVID-19, Neoadjuvant therapy, Surgical treatment, Adjuvant therapy, Treatment delay.

## Abstract

Breast cancer is the most common malignant tumor in women globally. Currently, due to limited data, there are no international guidelines for addressing the management of a large group of patients during infectious disease pandemics. Coronavirus disease 2019 (COVID-19), declared as a pandemic by the World Health Organization (WHO), has rapidly spread globally. The COVID-19 pandemic changed our daily routines and forced us to rethink the management of breast cancer patients. Clinicians need to take into account multiple factors such as the timing and delivery of cancer care, epidemic prevention and control, and the allocation of medical resources. Determining ways to reasonably adjust the treatment strategy is a real challenge. In this review, we aim to discuss particular challenges associated with managing breast cancer patients during the COVID-19 pandemic, share experience from Chinese oncologists and surgeons and propose some practical approaches to the management of early-stage breast cancer patients from a surgical standpoint.

## Introduction

The emergence of COVID-19 has caused a global public health emergency. In December 2019, an outbreak of respiratory disease caused by a novel coronavirus first emerged and has now spread to more than 150 counties 1-3. COVID-19 is characterized by rapid human-to-human transmission and has achieved pandemic spread 4-6. However, there are limited reports regarding the care of cancer patients during an infectious pandemic.

Breast cancer ranks first in the incidence of female malignant tumors in the world, and new patients with cumulative follow-up and adjuvant and salvage treatment continue to accumulate 7. Since the beginning of 2020, the rapid spread of the COVID-19 pandemic and the implementation of isolation policies have had a great impact on normal medical activities 8. For breast cancer, the allocation of medical resources and disease prevention and control largely affect the standardized treatment of breast cancer patients. Determining ways to maximize the benefits while balancing COVID-19 control and cancer management is very important at present.

Accumulating evidence has suggested that cancer patients are at higher risk of COVID-19 infection and are more likely to have higher morbidity and mortality than the general population 9-11. Compared with the general population, cancer patients had a two-fold increased risk of COVID-19 infection 10. The infection rate of SARS-CoV-2 in patients with cancer was 0.79% (95% CI = 0.3-1.2), higher than the cumulative incidence of all diagnosed COVID-19 cases 12. While many analyses from different counties are preliminary and require validation from larger international cohorts, several factors could account for the elevated risk of acquiring COVID-19 and consequential complications among cancer patients, including frequent hospital visits and admissions, immunocompromised state, advanced age, and poor functional status 10-12. Given the various problems in the diagnosis and treatment of breast cancer during pandemic, this paper aims to review the related clinical studies and guidelines for the diagnosis and treatment of breast cancer, and propose relevant suggestions for the management of early-stage breast cancer patients. Medical decision-making, such as the timing and approach of surgery, neoadjuvant therapy and adjuvant therapy, has a greater bearing on the outcomes of early-stage patients than those of late-stage patients. Here, we discussed the principles of early-stage breast cancer management, general diagnosis and treatment process, then the specific challenges and strategies regarding neoadjuvant therapy, surgical treatment and adjuvant therapy, and finally the issues associated with the delay of surgery and adjuvant therapy.

## Basic principles for the treatment of early-stage breast cancer patients under the COVID-19 pandemic

During the pandemic period of COVID-19, the principles of breast cancer treatment should be made scientifically and adjusted reasonably. If breast cancer patients present with a COVID-19 coinfection, the treatment should have an anti-pneumonia focus, and the patients should be admitted to a COVID-19-designated hospital 13. Serious patients should have anti-tumor treatments suspended and be actively treated for COVID-19. For mild infections, breast cancer patients who are undergoing endocrine therapy can maintain single-drug endocrine therapy if appropriate. For these patients, a delay in treatment for breast cancer of several weeks would not affect the overall efficacy. Later, the issue related to delayed treatments of breast cancer will be further explored.

The treatment of breast cancer should be based on the principle of nearby treatment 13, 14. This enables patients to avoid intercity travel and reduce the risk of infection with COVID-19. With the homogenization of the level of diagnosis and treatment in various places, hospitals at all levels can provide effective treatment for the majority of breast cancer patients, and patients can also obtain references and suggestions from breast cancer centers through online consultation.

For patients with difficulties in obtaining systemic treatment, we may make corresponding adjustments in the stages of adjuvant therapy, neoadjuvant therapy and even rescue therapy. For patients with difficulties in coming to the hospital, oral drugs are preferred 13, 14. Priority should be given to oral endocrine therapy or oral chemotherapeutic drugs, mainly administered at home 13-16. For patients who can be admitted to the hospital, short-term infusion and treatment with relatively low toxicity and side effects are recommended. PEG-rhG-CSF (pegylated recombinant human granulocyte colony-stimulating factor) can be used prophylactically in the chemotherapy cycles. For patients using adjuvant GnRH (gonadotropin releasing hormone) agonists to optimize ovarian function suppression, 3-month GnRH agonist administration is recommended to reduce the number of visits to the hospital. The Chinese Medical Insurance Bureau has introduced a long prescription policy, which can reduce the number of patients coming to the hospital to receive medicine.

## Diagnosis and treatment processes of early-stage breast cancer patients under the COVID-19 pandemic

**Figure 1:**
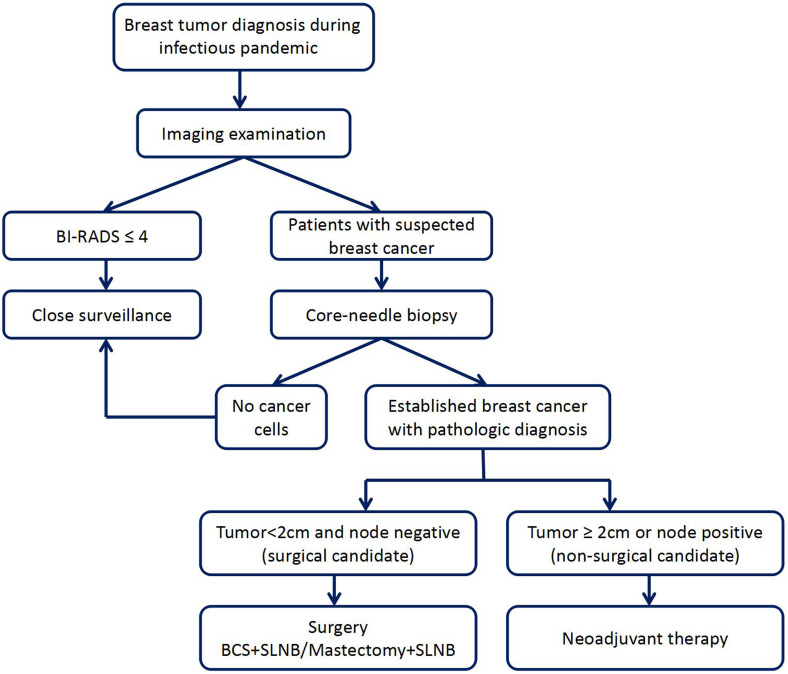
The suggested approach to breast tumor diagnosis and treatment decisions during an infectious disease pandemic.

Under the COVID-19 pandemic, patients should make an appointment to see a doctor in the hospital to ensure safety and protection from the disease and undergo specialized breast examination after COVID-19 screening. First, patients should undergo imaging examination, mainly mammography and ultrasonography; some patients may also need MRI examination. If the imaging classification is less than BI-RADS 4, we recommend regular follow-up and re-examination for 3 to 6 months 17, 18. For patients whose masses are diagnosed as BI-RADS 4 and 5, core-needle biopsies are preferred. If lymph node metastasis is considered on clinical evaluation, lymph node biopsy should be performed at the same time. We will specifically stratify patients whose core-needle biopsy indicates breast cancer. Patients with tumor diameters less than 2 cm and node-negative can be considered for receiving surgical treatment directly 13. Breast-conserving surgery and sentinel lymph node biopsy are recommended, which may allow the implementation of local anesthesia, faster postoperative recovery and short hospital stay 15, 16. Patients with larger tumor or lymph node metastasis are recommended to choose appropriate preoperative neoadjuvant therapy 13-16. The suggested approach to breast tumor diagnosis and treatment decisions during an infectious disease pandemic, including oncological surgical consideration, is shown in Figure 1. However, the approach should also be adjusted according to the specific conditions of the patients.

## Neoadjuvant therapy of early-stage breast cancer patients under the COVID-19 pandemic

According to the guidelines for breast cancer diagnosis and treatment, the indications for neoadjuvant therapy for breast cancer include stage II or stage III TNBC (triple-negative breast cancer) or HER2 (human epidermal growth factor receptor 2)-positive breast cancer, stage II or stage III HR (hormone receptor)-positive breast cancer that is expected to undergo adjuvant chemotherapy, and certain breast cancer patients who need better surgical treatment, i.e., patients who require shrinking of the tumor to be able to undergo breast-conserving surgery, those who need to achieve pathological complete response of the axillary metastatic lymph nodes to achieve axilla preservation and those who need to reduce tumor burden to achieve R0 resection 19,20. It is recommended that patients who require neoadjuvant therapy be screened with the support of a breast cancer MDT (multidisciplinary teamwork) to ensure that patients who are more suitable for neoadjuvant therapy are selected and optimal neoadjuvant therapy is provided; the unavailability of a multidisciplinary team may prove challenging and risky 20.

During the COVID-19 pandemic, neoadjuvant therapy is recommended primarily for patients whose tumor diameters are larger than 2 cm or who are clinical node-positive. For breast cancer patients with small tumors who cannot receive surgical treatment within a limited time, the indications of neoadjuvant therapy can be appropriately broadened according to IHC (immunohistochemistry) results 13-16. The treatment principles should as much as possible follow high quality international clinical guidelines. However, some of the systemic therapies have a significant risk of immunosuppression that can have potential detrimental effects during the pandemic, and some measures should be taken to decrease the detrimental effects. To decrease the number of visits to the hospital, 3-weekly chemotherapy regimens should be preferred, and hematopoietic growth factors can be used to decrease the risk of neutropenia and febrile neutropenia, moreover, steroids use should be limited to the indispensable to avoid increasing the risk of immunosuppression, and the use of nab-paclitaxel should be considered in some regimens.

For HR-positive and HER2-negative breast cancer, neoadjuvant endocrine therapy should be considered first and aromatase inhibitors should be given priority, in addition, the use of ovarian function suppressants should be included in premenopausal women 13,16. For TNBC, when adding platinum to anthracyclines and taxanes, the higher hematological toxicity must be taken into account during the pandemic, and single agent chemotherapy can also be considered 13. For HER2-positive breast cancer, the use of anti-HER2 agents is highly recommended, and it is recommended that paclitaxel or docetaxel combined with dual trastuzumab and pertuzumab blockade regimens should be given priority, as it appears that this regimen is relatively safe 13, 21, 22. Patients who are undergoing neoadjuvant therapy should proceed as planned. However, the treatment scheme can also be adjusted according to the specific conditions of the patients and the local pandemic situation based on the opinions above, and treatment regimens with low toxicity and curative effects can be reasonably replaced.

Patients who have completed the assigned neoadjuvant regimens should undergo the designated operation within 4 weeks, but in this special situation, patients for whom the therapy was effective can delay the operation by 2 to 4 weeks 15, 16. For patients with effective neoadjuvant therapy but for whom breast surgery is temporarily unavailable, based on the possible postoperative adjuvant therapy, adjuvant regimens can be given in advance to maintain the consistency of treatment 13, 15, 16. HR-positive and HER2-negative breast cancer patients can be treated with endocrine therapy, while HER2-positive breast cancer patients can also stop chemotherapy and continue with any previous effective targeted therapies. Accordingly, patients who achieve pCR (pathological complete response) after completing the full cycles of the assigned neoadjuvant regimens should be continuously administered the previously effective targeted regimens, either trastuzumab or trastuzumab combined with pertuzumab, and patients who do not achieve pCR (non-pCR), should be treated with T-DM1 (ado-trastuzumab emtansine) after the operation according to the KATHERINE Trial 23. Patients with TNBC may consider oral capecitabine according to the CREATE-X Trail 24. For situations where operations are temporarily unavailable, priority should be given to low-toxicity, effective and easy-to manage systemic treatments, and surgical treatment should be performed when conditions permit.

## Surgical treatment of early-stage breast cancer patients under the COVID-19 pandemic

Currently, patients in China need to receive body temperature measurements, routine blood tests, lung CT, and IgG, IgM and nucleic acid testing for COVID-19 before admission for surgical treatment 11. We should adopt the principle of one patient in one room, strictly limit visits and control the overall number of patients. Surgical treatment should be reserved mainly for patients with operable breast cancer, especially certain patients with early-stage breast cancer, patients who intend to undergo surgery after neoadjuvant therapy, and certain patients with operable breast cancer undergoing rapid progression.

Breast cancer patients who have been diagnosed with or are suspected of novel coronavirus disease might postpone the administration of cancer treatment for safety reasons; especially patients who need to receive anti-coronavirus disease treatment, which is preferred. Patients who have contacted COVID-19 patients or suspected COVID-19 patients should receive lung CT, routine blood tests, IgG, IgM and nucleic acid testing, and isolate for 14 to 21 days 11. Patients will be transferred to the ward for treatment after it is confirmed that they are not infected. For patients with fever of unknown cause, it is necessary to identify the cause of fever and exclude COVID-19 infection before admission.

In this special period, surgery for benign breast tumors is postponed. For malignant breast tumors, we should attempt to perform a relatively minimal operation with quick recovery. Due to the limited medical resources, we should adjust the treatment strategy for early breast cancer accordingly. For patients with primary tumors less than 2 cm and who are node-negative, surgical treatment should be actively performed when local medical conditions permit, preferably breast-conserving surgery and SLNB (sentinel lymph node biopsy), and if the patients refuse breast-conserving surgery, mastectomy and SLNB can be performed 14, 15. Patients receiving these two kinds of surgery would have a shorter hospital stay and can even undergo ambulatory surgery. As the COVID-19 pandemic improves, some patients can also undergo immediate implant or expander reconstruction, but in areas where the pandemic has not been properly controlled, complex reconstruction surgeries are not recommended and should be delayed 15, 16.

For postoperative follow-up, most patients recover well after breast surgery with few major side effects. Wound care is simple and patients will be taught and given specific instructions in the management of various types of wound coverage and the drain care. However, there are still some patients may have postoperative complications, based on this, most hospitals in China have set up specific wound care clinics to deal with postoperative complications during the pandemic. Postoperative return appointments usually take place 7-10 days after the surgery date and should be scheduled with surgical team after surgery. During the return appointment, the surgical team will check the wound recovery, review the pathology report and discuss the next treatment steps with breast cancer patients.

## Adjuvant therapy of early-stage breast cancer patients under the COVID-19 pandemic

During the COVID-19 pandemic period, it is necessary to restrict the indications of adjuvant chemotherapy and avoid unnecessary chemotherapy. Oncologists should carefully weigh the advantages and disadvantages of chemotherapy, try to choose adjuvant regimens with a low risk of neutropenia, strictly calculate the dosage, and not exceed the standard recommended dose. Furthermore, TAC (docetaxel 75 mg/m^2^ IV day 1, doxorubicin 50 mg/m^2^ IV day 1, cyclophosphamide 500 mg/m^2^ IV day 1, cycled every 21 days for 6 cycles) chemotherapy regimens and dose-dense AC (doxorubicin 60 mg/m^2^ IV day 1, cyclophosphamide 600 mg/m^2^ IV day 1, cycled every 14 days for 4 cycles) followed by paclitaxel or weekly paclitaxel regimens should be avoided 13. Postoperative chemotherapy can be postponed for 2 to 4 weeks, and PEG-rhG-CSF is recommended for primary prevention. If adjuvant chemotherapy is interrupted, oral chemo-drugs are recommended, such as vinorelbine or capecitabine. Endocrine therapy can be used after chemotherapy for HR-positive and HER2-negative breast cancer patients or directly used for low-risk patients who may avoid chemotherapy. For postmenopausal patients, aromatase inhibitors are preferred; for low-risk premenopausal patients, tamoxifen can be taken; for medium- or high-risk premenopausal patients, the combined use of GnRH agonists is recommended; if injections of GnRH agonists are temporarily unavailable, tamoxifen can be used alone 13,16. Patients with HER2-positive breast cancer should be prioritized for receiving single chemo-drugs combined with dual blockade, while according to the APT trial, patients with tumors smaller than 2 cm and who are node-negative should be given paclitaxel combined with trastuzumab 25,26. When targeted therapy is interrupted, if the interval between two consecutive transfusions is less than 6 weeks, the original dose (6 mg/kg transtuzumab and 420 mg pertuzumab) should be intravenously infused as soon as possible; if the interval is longer than or equal to 6 weeks, the loading dose (8 mg/kg trastuzumab and 840 mg pertuzumab) should be given again, followed by a maintenance dose (6 mg/kg trastuzumab and 420 mg pertuzumab) every 3 weeks for one year.

In principle, adjuvant radiotherapy should be completed within 6 months after the operation. However, under the COVID-19 pandemic situation, if patients are temporarily unable to receive radiotherapy, it may be postponed for 1 to 2 months, and the patients can preferentially receive adjuvant endocrine therapy or anti-HER2 targeted therapy.

## Delay of surgical treatment in early-stage breast cancer patients under the COVID-19 pandemic

Delayed surgery was a common problem for breast cancer patients. Both doctors and patients are concerned about whether the delayed treatment of breast cancer will affect the prognosis. Although there are no clear guidelines for the interval between diagnosis and treatment of breast cancer, through some retrospective studies, we briefly analyze the relationship between the interval time and the survival of breast cancer patients.

A number of retrospective studies showed that an interval between diagnosis and initial treatment of less than 30 days had no significant effect on OS (overall survival) and DFS (disease-free survival) in breast cancer patients, and an interval between 60 to 90 days had no significant effect on OS; however, a delay of more than 12 weeks might significantly affect the prognosis of breast cancer patients 27-29. Several retrospective analyses based on large clinical data showed that OS decreased significantly as the interval between diagnosis and initial treatment increased beyond 30 days for stage I and stage II breast cancer patients, and BCSS (breast cancer-specific survival) decreased significantly as the interval increased beyond 60 days only in stage I patients 30-33. Furthermore, a surgery delay of 90 days was significantly correlated with a decrease in OS in stage I and stage II patients 28, 31. According to the above studies, patients who meet the surgical conditions after diagnosis can appropriately postpone surgical treatment, and a delay of less than 90 days will not significantly affect OS. Therefore, during the COVID-19 pandemic, 90 days can be used as a reasonable limit for breast cancer patients to postpone surgery. However, for early-stage breast cancer, especially stage I, if conditions permit, surgical treatment should be performed within 30 days.

Another important issue is the management of patients who have currently completed neoadjuvant therapy and are waiting for surgery. Although many studies have suggested that delayed surgical treatment could lead to poor prognosis in breast cancer patients, the delay between neoadjuvant therapy and surgery has been ignored by many prospective clinical trials of neoadjuvant chemotherapy 31, 34. At present, the physical condition of patients receiving neoadjuvant therapy needs 2 to 3 weeks to recover from the toxic reactions of neoadjuvant regimens, therefore surgery is typically performed during this time. However, the influence of delayed surgery on prognosis of breast cancer patients was unclear. Retrospective studies showed that an interval of less than 3 weeks tended to improve the prognosis of breast cancer patients, and an interval of less than 6 weeks did not affect RFS (relapse-free survival), LRFS (locoregional recurrence-free survival) or OS in breast cancer patients 34-36. Therefore, patients receiving neoadjuvant chemotherapy should undergo surgery as soon as possible. Patients who have completed standard neoadjuvant chemotherapy and are waiting for surgery should be operated upon as soon as possible if the clinical evaluation reaches CR (complete response), and in this special period, the interval between neoadjuvant and surgery can be extended to 6 weeks. If the efficacy evaluation does not reach CR, the follow-up treatment plan can be adjusted for the patients according to the clinical evaluation effect.

## Delay of adjuvant therapy in early-stage breast cancer patients under the COVID-19 pandemic

The timing of adjuvant chemotherapy after breast cancer surgery is still controversial. The guidelines of the European Society of Oncology (ESMO) recommend that the best time for adjuvant chemotherapy is 2 to 6 weeks after surgery. The American Society of Clinical Oncology (ASCO) recommended that patients receive adjuvant chemotherapy no more than 120 days from the diagnosis of breast cancer. Although some studies suggest that the short-term delay of adjuvant chemotherapy will not affect survival, many studies have indicated as delay time increases, the benefit of adjuvant chemotherapy is reduced, affecting the prognosis of breast cancer patients 37. Several retrospective studies showed that a delay of adjuvant therapy of more than 12 weeks significantly reduced DFS and OS in breast cancer patients 37-40. Delayed adjuvant chemotherapy may have different effects on the prognosis of breast cancer patients with different stages or molecular types. Studies have found that in patients with TNBC and HER2-positive breast cancer treated with trastuzumab, the decrease in DFS and OS was significant when adjuvant therapy was delayed for more than 60 days but not in HR-positive and HER2-negative breast cancer patients 40, 41. During the COVID-19 pandemic, adjuvant chemotherapy for postoperative breast cancer patients could be appropriately postponed, but it should not exceed 12 weeks after surgery. When ongoing adjuvant chemotherapy has to be interrupted, this postponement may have a certain impact on prognosis. Theoretically, the delay in adjuvant chemotherapy may lead to tumor recurrence and drug resistance, especially in patients with late-stage breast cancer and high sensitivity to chemotherapy. At present, there are few studies on the delay of adjuvant therapy. Due to the lack of sufficient evidence, we recommend that systemic adjuvant therapy should be resumed as soon as possible if the condition permits.

Under the COVID-19 pandemic, patients with HR-positive breast cancer in some areas may face interruption or delay of adjuvant endocrine therapy. Retrospective analyses of compliance with endocrine therapy for breast cancer patients have indicated that early withdrawal or poor compliance of postoperative adjuvant endocrine therapy can increase the risk of recurrence and death, while insisting on endocrine therapy would achieve better long-term survival 42,43. During the COVID-19 pandemic, the Chinese Health Insurance Bureau has made long-prescription adjustments to provide protection for breast cancer endocrine therapy, including aromatase inhibitors and SERMs (selective estrogen receptor modulators). Short-term withdrawal of endocrine medicine for 2-4 weeks will not affect the prognosis of breast cancer patients, and we can strengthen the management of endocrine therapy by adjusting the health insurance policy and drug delivery in this special period.

HER2-positive patients in some areas may face an interruption or delay of adjuvant targeted therapy. Although the best time for starting adjuvant trastuzumab treatment is still unclear, the HERA trial showed that the therapeutic effect of 2-year trastuzumab is not better than that of 1-year treatment, indicating that the best treatment period may be within 12 months 44. Retrospective studies showed that patients who received trastuzumab more than 6 months after diagnosis had a higher risk of recurrence and death than those who received treatment sooner, and patients whose interval from diagnosis to the first treatment with trastuzumab was less than 12 weeks had significantly better DFS and OS 45, 46. For early-stage HER2-positive breast cancer patients, anti-HER2 targeted therapy within 6 months after surgery is the optimal strategy, and the longer the delay in starting adjuvant therapy is the worse the DFS and OS, so targeted therapy should be started as soon as possible 47, 48. For patients who are forced to discontinue targeted treatment with trastuzumab or trastuzumab combined with pertuzumab, the maintenance dose should be given immediately if the treatment interval is less than 6 weeks; for patients with a treatment interval greater than or equal to 6 weeks, the initial dose should be given again to make up for the missed treatment, and 1-year targeted therapy is still the current standard.

The impact of radiotherapy delay is different for patients with different stage or various surgeries. Retrospective studies showed that an interval of more than 12 weeks between surgery and radiotherapy resulted in a significantly higher risk of IBCR (ipsilateral breast cancer recurrence) in patients with DCIS (ductal carcinoma *in situ*) who underwent breast-conserving surgery, and an interval of more than 12 weeks led to poor DFS and OS in patients with early-stage breast cancer who underwent breast-conserving surgery 49, 50. Moreover, several studies found that an interval of less than 20 weeks did not affect IBCR, LRFS and BCSS in patients with stage I-III breast cancer 51-53. Karlsson et al retrospectively studied 964 breast cancer patients who received breast-conserving surgery and adjuvant endocrine therapy, and the results indicated that an interval between surgery and radiotherapy of less than 20 weeks did not affect DFS and OS 54. Therefore, in the interest of alleviating current workloads and resource constraints, evidence exists to support delaying radiation therapy among certain populations, as follows:

***DCIS*:** In patients requiring radiotherapy for DCIS, radiation can be safely delayed up to 12 weeks after breast-conserving surgery.

***Invasive breast cancer:*** Patients with early-stage, node-negative, HR-positive breast cancer can safely begin radiotherapy 8 to 12 weeks after breast-conserving surgery without compromising disease control or survival, with several large studies showing that a delay of up to 20 weeks may be safe in an appropriate subset. There is limited evidence to guide the interval from chemotherapy to radiotherapy and extrapolation from the aforementioned literature suggests that an interval of up to 12 weeks from chemotherapy to radiotherapy may be reasonable 53-55.

Under extreme circumstances, it may be necessary to prioritize which patients with breast cancer can receive radiotherapy services. Prioritization of patients for whom radiotherapy is anticipated to provide a survival benefit is paramount. Based on available evidence and nascent clinical judgment, we have defined tiers of elevated priority, with prioritization made based on patient age, comorbidity, risk of exposure, and predicted benefit of radiotherapy.

## Conclusions

In conclusion, under the COVID-19 pandemic, breast oncologists should make scientific decisions and reasonable adjustments to minimize its impact on breast cancer treatment. Moreover, breast oncologists should protect patients from COVID-19 infection during breast cancer treatment and ensure the continuity of breast cancer treatment to the greatest extent. All treatment decisions should be made based on a multidisciplinary tumor board, and all treatment decision-making should balance the risks and benefits of treatment in the context of a specific pandemic level on a case-by-case discussion, always including patient preferences. Here we summarize the effective managements of breast cancer patients accumulated by Chinese breast oncologists during the COVID-19 pandemic and to provide guidelines according to the pandemic scenario in each country on how to triage, prioritize and develop diagnostic procedures, surgical, radiation and medical treatments for early-stage breast cancer patients.

## Abbreviations

COVID-19: Coronavirus disease 2019
WHO: World Health Organization
PEG-rhG-CSF: pegylated recombinant human granulocyte colony-stimulating factor
GnRH: gonadotropin releasing hormone
TNBC: triple-negative breast cancer
HER2: human epidermal growth factor receptor 2
HR: hormone receptor
MDT: multidisciplinary teamwork
IHC: immunohistochemistry
pCR: pathological complete response
T-DM1: ado-trastuzumab emtansine
OS: overall survival
DFS: disease-free survival
BCSS: breast cancer-specific survival
RFS: relapse-free survival
LRFS: locoregional recurrence-free survival
CR: complete response
ESMO: European Society of Oncology
ASCO: American Society of Clinical Oncology
SERM: selective estrogen receptor modulator
IBCR: ipsilateral breast cancer recurrence
DCIS: ductal carcinoma *in situ*
